# Role of the Lymphocyte Count-to-C-Reactive Protein Ratio in the Risk Stratification for High EASE Scores After Living Donor Liver Transplantation: A Retrospective Observational Cohort Study

**DOI:** 10.3390/jcm13237344

**Published:** 2024-12-02

**Authors:** Jaesik Park, Chul Soo Park, Min Suk Chae, Ho Joong Choi, Sang Hyun Hong

**Affiliations:** 1 Department of Anesthesiology and Pain Medicine, Seoul St. Mary’s Hospital, College of Medicine, The Catholic University of Korea, Seoul 06591, Republic of Korea; 2 Department of Surgery, Seoul St. Mary’s Hospital, College of Medicine, The Catholic University of Korea, Seoul 06591, Republic of Korea

**Keywords:** early allograft failure, EASE score, early allograft dysfunction, living donor liver transplantation, end-stage liver disease, postoperative complications

## Abstract

**Background:** Early allograft failure (EAF) significantly contributes to mortality, necessitating re-transplantation following liver transplantation. The EAF simplified estimation (EASE) score has been recently developed to predict EAF. We aimed to assess the predictive capacity of high EASE scores for EAF and postoperative outcomes and to evaluate the association between the lymphocyte count-to-C-reactive protein ratio (LCR) and high EASE scores after living donor liver transplantation (LDLT). **Methods:** We retrospectively analyzed the data of 808 patients who underwent LDLT. After excluding 16 patients with incomplete laboratory data, the final cohort included 792 patients. Patients with EASE scores ≥−0.74 were categorized into the high EASE group. Multivariate logistic regression was used to examine the association between the LCR and high EASE scores. **Results:** High EASE scores demonstrated superior predictive accuracy for EAF development relative to that of the early allograft dysfunction (EAD) model (*p* = 0.018) and were more closely associated with overall mortality (*p* = 0.033). A preoperative LCR < 12.7 significantly increased the odds (odds ratio, 3.3; confidence interval, 1.997–5.493) of exhibiting high EASE scores post-LDLT, alongside preoperative hematocrit levels, operative duration, intraoperative continuous renal replacement therapy, administered calcium dose, mean heart rate, and donor age. **Conclusions:** The EASE score could offer enhanced utility for predicting EAF and overall mortality following LDLT relative to that of EAD. Identifying and managing risk factors, including low LCR values, for elevated EASE scores is essential for improving patient prognoses.

## 1. Introduction

Living donor liver transplantation (LDLT) is a well-established curative intervention in patients with advanced liver disease [1]. Early allograft failure (EAF) refers to the loss of the graft through either re-transplantation or patient death within the initial three months post-transplantation [2,3]. Consequently, EAF following liver transplantation (LT) poses a significant clinical challenge, which impacts both graft and recipient survival. Olthoff et al. [4] introduced a widely recognized method for predicting EAF using the early allograft dysfunction (EAD) model, which considers postoperative levels of aspartate aminotransferase (AST), alanine aminotransferase (ALT), serum bilirubin, and international normalized ratio (INR). The EAF simplified estimation (EASE) score, a newly developed model, estimates EAF by incorporating perioperative factors such as the model for end-stage liver disease (MELD) score, volume of packed red blood cell (PRBC) units transfused during the procedure, presence of hepatic vessel thrombosis, and case volume of the transplant center (≥70 per year) [5]. The EASE score has demonstrated high predictive value for estimating EAF after LT and has proven superior to the EAD model [5,6]. Therefore, early identification of patients with high EASE scores is essential for effective patient management and timely intervention. EASE risk groups for graft and patient survival range between 1 and 5, with higher numbers indicating increased risk. We considered risk groups 4 and 5 as high-risk and evaluated the predictive value of a high EASE score (≥−0.74) in relation to that using the EAD model.

A decreased total lymphocyte count helps predict the risk of postoperative complications owing to malnutrition [7]. Moreover, low lymphocyte counts correlate with the severity of liver cirrhosis [8]. C-reactive protein (CRP), an acute-phase inflammatory marker produced in the liver, manifests elevated levels in advanced liver disease and correlates with increased mortality from systemic inflammation [9]. The lymphocyte count-to-CRP ratio (LCR) has been associated with symptom severity in patients with coronavirus disease 2019 [10] and exhibits predictive value for various clinical conditions, including cancer and myocardial infarction [11,12]. However, the relationship between LCR and morbidity following LT remains unexplored.

Therefore, we investigated the predictive value of a high EASE score in estimating EAF and other outcomes, including acute kidney injury (AKI) and overall mortality. Moreover, we examined the risk factors for high EASE scores using multivariate logistic analysis.

## 2. Patients and Methods

### 2.1. Study Population

Data of 808 adult patients (aged over 19 years) who underwent elective LDLT at Seoul St. Mary’s Hospital from January 2009 to July 2022 were retrospectively collected using the electronic medical record system. The inclusion criteria included adult patients (age ≥ 19 years) undergoing primary LDLT. The exclusion criteria included cases of incomplete data resulting from death before postoperative day (POD) 10 or other causes. After these exclusions, the final cohort comprised 792 adult patients.

### 2.2. Living Donor Liver Transplantation

A team of expert surgeons and anesthesiologists performed transplantation surgery and administered general anesthesia. The transplantation procedure was performed under general anesthesia by an experienced team of surgeons and anesthesiologists. Using the piggyback technique, which preserved the recipient’s inferior vena cava, the right lobe of the liver was transplanted. Following the completion of the vascular and ductal connections, Doppler ultrasonography was used to confirm proper hepatic blood flow, such as portal venous flow and the hepatic artery resistive index. Interventions such as splenectomy or portocaval shunt were applied, if necessary. General anesthesia was adjusted for maintaining stable hemodynamics, ensuring that the mean blood pressure (MBP) was kept at or above 65 mmHg, and the central venous pressure (CVP) remained at or below 10 mmHg. Transfusion practices were employed to ensure adequate blood component supply and to stabilize vital signs [13]. PRBCs were administered to maintain a hematocrit level ≥25%. Furthermore, coagulation factors such as fresh frozen plasma (FFP), single donor platelets, and cryoprecipitate were provided based on laboratory test results or thromboelastography findings.

Patients experiencing severe preoperative kidney function loss underwent continuous renal replacement therapy (CRRT) during surgery [14,15]. Profound postreperfusion syndrome was identified by a decrease in MBP by ≥30% compared with that of the anhepatic phase, hypotension lasting ≥5 min, severe arrhythmias, or the need for vasopressors (epinephrine or norepinephrine).

Immunosuppression was managed according to the LDLT protocol of our institution, which included administration of basiliximab, calcineurin inhibitors, mycophenolate mofetil, and prednisolone, as well as postoperative tapering of immunosuppressants.

### 2.3. Early Allograft Failure Simplified Estimation Score

The EASE score was calculated using the following formula [16]: EASE = −0.602 + 0.044 × (MELD score at transplantation) + 0.065 × (number of transfused PRBC units during the procedure) + 2.567 (occurrence of hepatic vascular thrombosis during postoperative days [PODs] 1–10) + 0.000534 × (area under the receiver operating characteristic curve [AUC] for AST on PODs 1, 2, 3, 7, and 10)^2^ − 0.093 × (AUC for platelet count on PODs 1, 3, 7, and 10)–7.766 × (slope for platelet count on PODs 1, 3, 7, and 10) + 0.795 × (slope for bilirubin on PODs 1, 3, 7, and 10) − 0.402 (if annual center volume ≥70 cases). Risk groups were defined as follows: group 1: EASE score < −3.43, group 2: −3.43 ≤ EASE score ≤ −1.26, group 3: −1.25 ≤ EASE score ≤ −0.75, group 4: −0.74 ≤ EASE score ≤ −0.01, and group 5: 0 ≤ EASE score ≤ 5. Groups 4 and 5 were considered high-risk groups.

### 2.4. Early Allograft Failure

EAF was defined as a graft failure that resulted in death or the need for re-transplantation for any reason by 3 months after surgery [2,3].

### 2.5. Early Allograft Dysfunction

EAD was diagnosed if one or more of the following postoperative laboratory findings were present: total bilirubin ≥ 10 mg/dL on POD 7, an INR ≥ 1.6 on POD 7, or ALT or AST levels exceeding 2000 IU/L within the first seven PODs [4,17].

### 2.6. Lymphocyte and CRP Measurements

Laboratory parameters were measured using blood samples collected in tubes with either ethylenediaminetetraacetic acid (EDTA) as an anticoagulant (BD Vacutainer, K2 EDTA; Becton, Dickinson and Company, Franklin Lakes, NJ, USA) or with clot activator in a serum separator tube (BD Vacutainer, Becton, Dickinson and Co., Franklin Lakes, NJ, USA). Lymphocyte counts were measured in samples collected in EDTA-coated tubes, while CRP levels were measured in samples in serum clot activator tubes.

LCR = lymphocyte count (per µL)/CRP (mg/dL)/100.

The optimal cutoff value of LCR (<12.7) in predicting a high EASE score was identified using area under the curve analysis (AUC: 0.705; 95% confidence interval [CI]: 0.672–0.737; *p* < 0.001).

### 2.7. Recipient and Graft-Donor Variables

The study included variables such as sex, age, body mass index (BMI), underlying causes of end-stage liver disease, MELD score, diabetes mellitus, hypertension, hepatic decompensation (e.g., varices and ascites), and echocardiographic parameters (ejection fraction and presence of diastolic dysfunction). Varices were confirmed by either preoperative esophagogastroduodenoscopy or a history of variceal bleeding, while ascites was confirmed by CT or a history of ascites tapping. To assess left ventricular ejection fraction, we used the modified Simpson method, measuring the apical four-chamber and apical two-chamber views at end diastole and end systole. Diastolic dysfunction was diagnosed if at least three of the following criteria were met: (1) medial e’ < 7 cm/s (or lateral e’ < 10 cm/s); (2) average E/e’ ≥ 15; (3) left atrial volume index > 34 mL/m^2^; or (4) tricuspid regurgitation velocity > 2.8 m/s.

The laboratory variables included hematocrit, LCR, total bilirubin, INR, albumin level, platelet count, and white blood cell count, as well as calcium, glucose, creatinine, sodium, ammonia, and potassium concentrations, which were all assessed. The intraoperative variables included operative time, intraoperative CRRT, mean heart rate, MBP, CVP, occurrence of postreperfusion syndrome, total amount of calcium infused, blood product transfusion (PRBC, FFP, and PC) fluid infusion rate per hour, and urine output per hour. Donor-graft variables included graft-to-recipient weight ratio, the percentage of fatty change, the ischemic time of the graft, donor age, and sex.

Outcomes after surgery included overall patient mortality, AKI, and graft rejection [18].

### 2.8. Statistical Analysis

The perioperative findings were analyzed using the Chi-squared (χ^2^), Fisher’s exact, or Mann–Whitney U tests. The predictive values of the EASE score, a high EASE score, EAD for EAF occurrence, and postoperative outcomes were compared using the AUC method and Delong’s test. The relationship between the perioperative findings and a high EASE score was examined through both univariable and multivariable logistic regression analyses. After univariate logistic analysis, factors with possible significance (*p* < 0.1) were fitted into multivariate logistic regression analyses, conducted using both forward and backward selection methods. Laboratory findings are presented as numbers (percentages) or medians (interquartile ranges [IQRs]). Statistical significance was set at *p* < 0.05. SPSS for Windows (version 24.0, SPSS Inc., Chicago, IL, USA) and MedCalc Statistical Software, version 23.0.2 (MedCalc Software bv, Ostend, Belgium), were used for these analyses.

## 3. Results

### 3.1. Patient Characteristics

The study cohort consisted primarily of men, accounting for 70.2% of the population. The median age, BMI, and MELD scores were 54 years (IQR: 48–60), 24.1 kg/m^2^ (IQR: 21.9–26.6), and 14 points (IQR: 7–24), respectively. The etiologies of liver disease were hepatitis B (54.2%), alcoholic liver disease (22.3%), hepatitis C (6.7%), cryptogenic disease (6.7%), hepatitis A (4.3%), autoimmune disease (4.2%), and drug-induced or toxic disease (1.6%). The median EASE score and LCR were −3.0 (IQR: −3.9–−1.7) and 19.4 (IQR: 4.9–80.0), respectively.

### 3.2. Comparison of the Prevalence of a High EASE Score and EAD

The incidence of EAF at 3 months after LDLT was 44 of 792 (5.5%). In patients with EAF, the number of patients with a high EASE score and EAD was 31 (70.5%) and 22 (50%), respectively (Table 1).

### 3.3. Comparison of the Predictive Value of the EASE Score, High EASE Score, and EAD for EAF Development

**Figure 1:**
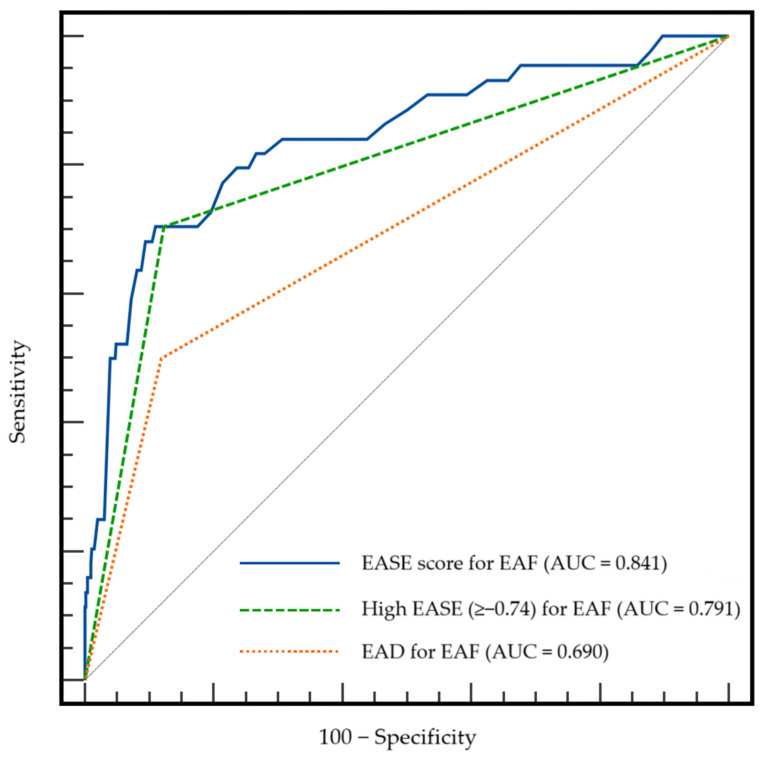
Comparison of AUCs among the EASE score, a high EASE score, and EAD for EAF. AUC, area under the receiver operating characteristic curve; EAD, early allograft dysfunction; EAF, early allograft failure; EASE, early allograft failure simplified estimation.

We compared the AUCs for EAF among the EASE score, a high EASE score, and EAD (0.841, 0.791, and 0.690, respectively; Figure 1). According to Delong’s method, both the EASE score and a high EASE score showed a significantly better predictive ability than did the EAD (*p* < 0.001 and 0.018, respectively).

### 3.4. Comparison of the Predictive Accuracy of a High EASE Score and EAD for Postoperative Outcomes

We compared the AUCs for postoperative outcomes of a high EASE score and EAD. A high EASE score showed significantly better predictive value for overall patient mortality than did the EAD (*p* = 0.033). However, AUCs for AKI, graft rejection, infection, and graft failure were not significantly different (*p* = 0.185, 0.798, 0.233, and 0.148, respectively; Table 2). The overall mortality rate was 14% (111 of 792 patients), with a median follow-up duration of 5.4 years (IQR: 2.9–7.6 years); the follow-up duration ranged from 10 days to 12 years. The primary cause of death after LT was infection, with an incidence of 8.5%.

Regarding 1-year and 5-year mortality, the AUCs for high EASE and EAD scores were 0.684 vs. 0.621 and 0.650 vs. 0.593, respectively, indicating that early mortality was more accurately predicted.

### 3.5. Comparison of Perioperative Variables Between Low and High EASE Groups

Preoperative recipient variables, including the hematocrit, MELD score, ascites, LCR, platelets, creatinine, total bilirubin, and INR, were significantly different between the high and low EASE groups (Table 3).

Intraoperative and donor-graft variables, including intraoperative CRRT, operative time, calcium infusion dose, mean heart rate, blood product transfusion (PRBC, FFP, and platelet concentrate), hourly urine output, hourly fluid infusion, and donor age, were significantly different between the low and high EASE groups (Table 4).

### 3.6. Association of Perioperative Variables with a High EASE Score

Multivariate logistic regression analysis (Table 5) helped identify LCR as a significant predictor of high EASE scores, along with factors including preoperative hematocrit, operative time, intraoperative CRRT, calcium infusion dose, mean heart rate, and donor age. The model had an AUC of 0.803 (95% CI: 0.774–0.831), with a sensitivity of 75.8% and a specificity of 72.6% (*p* < 0.001). Furthermore, patients with a low LCR were more than three times as likely to develop EAD than were those with a high LCR (odds ratio [OR]: 3.31; 95% CI: 1.997–5.493; *p* < 0.001). Compared with the AUC of the multivariate logistic regression model without LCR (0.786), the model incorporating LCR demonstrated significantly improved performance, as indicated by Delong’s test (*p* = 0.0033).

### 3.7. Comparison of LCR Levels in the Low and High EASE Groups

Patients in the high EASE group exhibited a lower median LCR of 5.3 (IQR: 2.2–20.0) relative to 26.1 (IQR: 6.0–93.5) in the low EASE group.

### 3.8. The Predictive Accuracy of LCR for EAF

The predictive value of LCR for EAF showed an AUC of 0.730 (95% CI: 0.698–0.761), with a sensitivity of 75% and a specificity of 65% (*p* < 0.001).

### 3.9. Association Between a Low LCR and Inflammatory Factors

We found a significant association between a low LCR and several inflammatory factors, including white blood cell count, albumin concentration, and CRP level (all, *p* < 0.001).

### 3.10. Association of Perioperative Variables with EAF

Using univariate analysis, the following variables were identified with a *p*-value < 0.1 and fitted into the multivariate analysis: preoperative BMI, ejection fraction, MELD score, ascites, creatinine, intraoperative CRRT, operative time, MBP, mean heart rate, mean CVP, total calcium infusion, hourly fluid infusion, hourly urine output, graft-recipient-weight ratio, and total ischemic time. Using multivariate analysis, BMI, operative time, intraoperative CRRT, and MBP were significantly associated with EAF. The model achieved an AUC of 0.753 (95% CI: 0.722–0.783), with a sensitivity of 75.0% and a specificity of 68.9% (*p* < 0.001).

### 3.11. Comparison of the Predictive Accuracy of Other Clinical Risk Scores for EAF

The predictive value of MELD, the balance of risk (BAR) score, and the liver graft assessment following transplantation (L-GrAFT) 7 for EAF were evaluated, revealing AUC values of 0.696, 0.697, and 0.806, respectively [2,3].

## 4. Discussion

Our findings indicated that a high EASE score was a more accurate predictor of EAF and other outcomes following LDLT than was the previously used EAD model. Moreover, a low LCR independently tripled the likelihood of a high EASE score post-LDLT, alongside preoperative hematocrit, intraoperative CRRT, calcium infusion dose, mean heart rate, and donor age.

Various factors influence outcomes after transplantation. Recipient and donor characteristics, as well as intra- and postoperative complications, can exacerbate graft dysfunction, ultimately leading to graft failure and death. Graft injury frequently results from ischemia and reperfusion injury (IRI) during LT, with poor recovery of the liver graft typically commencing with severe IRI, followed by ongoing coagulopathy [19]. EAF is a major contributor to patient death and re-transplantation within three months post-transplantation. Consequently, early assessment of graft function is crucial. EAD adversely affects outcomes after LT, leading to kidney damage, prolonged hospitalization, early liver graft failure, and increased mortality [4,17,20,21]. Several risk estimation models have been newly developed, including the recently introduced EASE score for EAF [5,6]. In previous studies, the EASE score showed better predictive value for EAF than did other prediction models, such as EAD. This is consistent with our finding that the EASE score was a better predictor of EAF than was EAD. We regarded the EASE score groups 4 and 5 as comprising the high EASE group (EASE score ≥−0.74), and a high EASE score also showed a higher predictive value than did EAD for both EAF and overall mortality. We also analyzed the predictive value of different scores, such as BAR and L-GrAFT 7, for EAF and found that the EASE score was the most accurate.

As prognostic markers, both lymphocyte count and CRP level have been extensively studied in various clinical contexts, including sepsis, cancer, and critical illness following ICU admission. A decreased lymphocyte count, often associated with advanced liver disease [8], possibly results from lymphocytes being recruited to the liver due to necroinflammation. Intrahepatic CD8^+^ T cells are significantly increased in patients with acute liver failure than the rate noted in healthy individuals [22]. Berres et al. reported that low lymphocyte counts were linked to higher mortality in patients with end-stage liver disease [23]. CRP, an acute-phase inflammatory marker synthesized primarily by the liver under conditions such as infection, trauma, and ischemia, increases in response to proinflammatory cytokines, particularly interleukin-6 [24,25,26,27,28,29,30,31,32]. Elevated CRP levels, observed during complex surgeries and in conditions such as sepsis and decompensated heart failure [28,29,30,33], correlate with increased morbidity and mortality in patients with advanced liver disease [9,34,35,36,37,38,39] and are significantly associated with outcomes related to hepatic injury or extrahepatic organ dysfunction [9,40]. Although CRP is primarily produced in the liver, other cells, such as macrophages and reserved hepatocytes, also contribute to CRP production in response to elevated interleukin-6 levels [41,42,43]. Thus, elevated CRP levels combined with a low lymphocyte count may indicate the severity and progression of liver injury.

Recent research has supported the use of the LCR as a tool for estimating outcomes in critically ill patients [44,45,46,47,48,49]. In a previous study, a low LCR, or in other words, a high CRP-to-lymphocyte ratio, was an effective prognostic marker in patients with cirrhosis [50]. Despite emerging evidence on the usefulness of the LCR, its utility in patients with LDLT has been limited. In the present study, the LCR independently predicted high postoperative EASE scores. Additionally, incorporating the LCR significantly improved the accuracy of the predictive model, suggesting that LCR could be a valuable preoperative risk factor for identifying patients at higher risk. LCR was significantly associated with inflammatory parameters such as CRP, white blood cell count, and albumin. Therefore, the association between the LCR and a high EASE score might reflect exacerbated systemic inflammation.

Our findings align with those of previous studies that identified operative time, intraoperative CRRT, and donor age as significant predictors of graft failure after LT. Lee et al. suggested that the duration from incision to the anhepatic phase was linked to graft survival [51]. Patients requiring CRRT are more likely to develop acute liver failure [52]. Moreover, donor age was significantly associated with an increased risk of graft failure [53]. According to multivariable analysis, preoperative anemia is associated with a higher number of intraoperative blood transfusions and postoperative complications, as previously reported [54]. The intraoperative calcium requirement is also related to the amount of blood transfused [55], and as a hemodynamic variable, the intraoperative heart rate is associated with negative outcomes after major surgery [56]. Although most variables are not adjustable, selection of younger donors when feasible may help improve patient outcomes.

This study has some limitations. First, although the LCR reflects systemic inflammation, further studies are required to understand the pathophysiological mechanisms linking the LCR to high EASE scores. Second, differences between the conditions of living and deceased donor liver transplantations might have affected the predictive value of a high EASE score for EAF. Third, as a retrospective study, we were unable to control for all potential confounders. Fourth, we used a *p*-value threshold of 0.1, instead of 0.05, to select clinically relevant variables to be fitted in multivariate analysis. Although including relevant variables up to a *p*-value of 0.1 is common in previous medical literature [57], this less stringent threshold could be considered a limitation to our study. Finally, although clinical factors associated with high EASE scores were accounted for in the multivariable analysis, the potential for selection bias cannot be entirely ruled out; thus, the results should be interpreted with caution.

## 5. Conclusions

Our findings suggest that a high EASE score, i.e., groups 4 and 5 of the recently developed EASE score for LT, was more useful for predicting EAF and overall mortality than was EAD. Moreover, preoperative LCR was a significant predictor of a high EASE score post-LDLT, along with operative time, preoperative hematocrit, intraoperative CRRT, amount of calcium infusion, mean heart rate, and donor age. Consequently, using the readily available LCR might help to estimate the risk of a high EASE score and to evaluate the overall status of patients. Factors such as BMI, operative time, intraoperative CRRT, and MBP were also significantly associated with EAF. Risk factors for a high EASE score and EAF, including the LCR, should be identified early, and these factors should be closely monitored. Due to the presence of multiple confounders and limitations in the study design, further prospective studies are required to validate the utility of the EASE score in clinical practice, particularly in patients undergoing LDLT.

## Figures and Tables

**Table 1:** Comparison of the prevalence of a high EASE score and EAD in patients with EAF.

Group	No EAF	EAF	*p*
n	748	44	
High EASE Score	92 (12.3%)	31 (70.5%)	<0.001
EAD	89 (11.9%)	22 (50%)	<0.001

Abbreviations: EASE, early allograft failure simplified estimation; EAD, early allograft dysfunction.

**Table 2:** Comparison of the predictive accuracy of a high EASE score and EAD for postoperative outcomes.

Group	AUC of High EASE	AUC of EAD	*p*
AKI	0.521	0.547	0.185
Graft rejection	0.504	0.510	0.798
Infection	0.525	0.543	0.233
Graft failure	0.626	0.592	0.148
Overall patient mortality	0.645	0.591	0.033

Abbreviations: AUC, area under the curve; AKI, acute kidney injury; EASE, early allograft failure simplified estimation; EAD, early allograft dysfunction.

**Table 3:** Preoperative recipient variables in the low and high EASE groups.

Group	Low EASE	High EASE	*p*
n	669	123	
Age (years)	54 (49–60)	53 (44–60)	0.248
Sex (male)	468 (70%)	93 (75.6%)	0.205
Body mass index (kg/m^2^)	24 (22–27)	24 (21–27)	0.869
Etiology			
Alcohol	151 (22.6%)	26 (21.1%)	0.307
Hepatitis A	28 (4.2%)	6 (4.9%)	
Hepatitis B	367 (54.9%)	62 (50.4%)	
Hepatitis C	43 (6.4%)	10 (8.1%)	
Autoimmune	23 (3.4%)	10 (8.1%)	
Drug and Toxin	12 (1.8%)	1 (0.8%)	
Cryptogenic	45 (6.7%)	8 (6.5%)	
Comorbidity			
Diabetes mellitus	172 (25.7%)	37 (30.1%)	0.312
Hypertension	148 (22.1%)	22 (17.9%)	0.293
MELD score (points)	12 (6–22)	20 (13–32)	<0.001
Hepatic decompensation			
Varix	167 (25.0%)	36 (29.3%)	0.315
Ascites	299 (44.7%)	84 (68.3%)	<0.001
Cardiac function			
Ejection fraction (%)	64 (62–67)	64 (60–67)	0.148
Diastolic dysfunction	291 (43.5%)	44 (35.8%)	0.111
Laboratory variables			
Hematocrit (%)	30 (26–36)	26 (23–31)	<0.001
WBC count (×10^9^/L)	4.5 (3.0–6.8)	4.9 (2.7–9.9)	0.198
Lymphocyte to CRP ratio	26.1 (6.0–93.5)	5.3 (2.2–19.8)	<0.001
Platelet count	70 (48–109)	56 (39–82)	<0.001
Albumin (g/dL)	3.1 (2.7–3.6)	3.1 (2.7–3.4)	0.227
Total bilirubin	2.1 (0.8–9.5)	7.1 (1.7–25.2)	<0.001
International normalized ratio	1.4 (1.2–2.0)	1.8 (1.3–2.4)	<0.001
Sodium (mEq/L)	139 (135–141)	138 (134–141)	0.124
Potassium (mEq/L)	4 (3.7–4.3)	4 (3.5–4.4)	0.563
Calcium (mg/dL)	8.4 (8.0–8.8)	8.3 (7.9–8.8)	0.357
Glucose (mg/dL)	108 (92–138)	116 (89–149)	0.326
Creatinine (mg/dL)	0.8 (0.7–1.0)	1.0 (0.7–2.1)	<0.001
Ammonia (μg/dL)	93 (65–146)	93 (56–148)	0.475

Abbreviations: CRP, C-reactive protein; MELD, Model for End-stage Liver Disease; WBC, white blood cell. Note: values are medians (ranges) or numbers (percentages).

**Table 4:** Intraoperative recipient and donor-graft variables in the low and high EASE groups.

Group	Low EASE	High EASE	*p*
n	669	123	
Operative time (min)	480 (435–542)	525 (475–600)	<0.001
Intraoperative CRRT	54 (8.1%)	35 (28.5%)	<0.001
Postreperfusion syndrome	333 (49.8%)	65 (52.8%)	0.531
Average of vital signs			
MBP (mmHg)	76 (70–81)	76 (69–84)	0.678
HR (beats/min)	88 (80–97)	92 (84–102)	0.003
CVP (mmHg)	9 (7.5–10.5)	9.3 (7.1–11.7)	0.205
Calcium infusion dose (mg)	300 (0–788)	1050 (300–4080)	<0.001
Blood product transfusion (unit)			
Packed red blood cell	7 (4–11)	13 (8–22)	<0.001
Fresh frozen plasma	6 (4–10)	10 (7–18)	<0.001
Platelet concentrate	4 (0–6)	6 (0–12)	<0.001
Hourly fluid infusion (mL/kg/h)	11.2 (8.4–13.8)	12.8 (9.0–17.9)	0.001
Hourly urine output (mL/kg/h)	1.4 (0.8–2.2)	1.0 (0.2–1.7)	<0.001
Donor-graft variables			
Age (years)	33 (27–40)	35 (32–46)	<0.001
Sex (male)	422 (63.1%)	74 (60.2%)	0.539
GRWR (%)	1.2 (1.1–1.5)	1.2 (1.0–1.6)	0.991
Graft ischemic time (min)	90 (70–112)	90 (69–120)	0.578
Fatty change (%)	5 (1–5)	5 (1–5)	0.686

Abbreviations: CRRT, continuous renal replacement therapy; CVP, central venous pressure; GRWR, graft-recipient-weight ratio; HR, heart rate; MBP, mean blood pressure. Note: values are medians (interquartile ranges) or numbers (percentages), unless indicated otherwise.

**Table 5:** Association of pre- and intraoperative recipient and donor-graft variables with a high EASE score.

	Univariate Analysis				Multivariate Analysis			
	*β*	Odds Ratio	95% CI	*p*	*β*	Odds Ratio	95% CI	*p*
Preoperative recipient variables								
Age (years)	−0.014	0.986	0.967–1.005	0.140				
Sex (male vs. female)	−0.286	0.751	0.482–1.170	0.206				
Body mass index (kg/m^2^)	0.007	1.007	0.959–1.057	0.790				
Comorbidity								
Diabetes mellitus	0.218	1.243	0.815–1.897	0.313				
Hypertension	−0.266	0.767	0.467–1.259	0.294				
Hepatic decompensation								
Varix	0.218	1.244	0.812–1.905	0.315				
Ascites	0.980	2.665	1.770–4.013	<0.001				
Cardiac function								
Ejection fraction (%)	−0.032	0.968	0.926–1.012	0.151				
Diastolic dysfunction	−0.119	0.888	0.662–1.191	0.427				
Laboratory variables								
Hematocrit (%)	−0.081	0.922	0.893–0.952	<0.001	−0.050	0.952	0.916–0.989	0.011
WBC count (x 10^9^/L)	0.030	1.031	1.002–1.060	0.036				
Lymphocyte count-to-CRP ratio	<0.001	1.000	1.000–1.000	<0.001	<0.001	1.000	1.000–1.000	<0.001
Lymphocyte count-to-CRP ratio (<12.7) *	1.530	4.617	2.997–7.111	<0.001	1.198	3.312	1.997–5.493	<0.001
Albumin (g/dL)	−0.229	0.796	0.579–1.094	0.160				
International normalized ratio	0.060	1.070	0.975–1.157	0.170				
Sodium (mEq/L)	−0.002	0.998	0.977–1.018	0.818				
Potassium (mEq/L)	−0.078	0.925	0.670–1.278	0.637				
Calcium (mg/dL)	−0.019	0.981	0.772–1.247	0.875				
Glucose (mg/dL)	0.001	1.002	0.998–1.005	0.446				
Creatinine (mg/dL)	0.267	1.306	1.152–1.481	<0.001				
Ammonia (μg/dL)	−0.001	0.009	0.997–1.002	0.702				
Intraoperative recipient variables								
Operative time (min)	0.005	1.005	1.003–1.007	<0.001	0.005	1.005	1.003–1.007	<0.001
Interoperative CRRT	1.511	4.530	2.802–7.323	<0.001	1.191	3.219	1.893–5.721	<0.001
Postreperfusion syndrome	0.179	1.196	0.908–1.576	0.203				
Vital sign averages								
MBP (mmHg)	0.005	1.005	0.998–1.011	0.147				
HR (beats/min)	0.024	1.024	1.010–1.039	0.001	0.022	1.022	1.006–1.038	0.008
CVP (mmHg)	0.068	1.070	1.009–1.134	0.024				
Calcium infusion dose (mg)	0.001	1.001	1.000–1.001	<0.001	<0.001	1.000	1.000–1.001	<0.001
Hourly fluid infusion (mL/kg/h)	0.019	1.019	1.000–1.038	0.051				
Hourly urine output (mL/kg/h)	−0.385	0.681	0.556–0.833	<0.001				
Donor-graft variables								
Age (years)	0.023	1.024	1.007–1.041	0.006	0.036	1.037	1.017–1.057	<0.001
Sex (male)	0.123	1.131	0.763–1.677	0.539				
GRWR (%)	0.222	1.248	0.850–1.834	0.258				
Graft ischemic time (min)	0.001	1.001	0.999–1.004	0.309				
Fatty change (%)	−0.005	0.995	0.964–1.028	0.765				

Abbreviations: CI, confidence interval; WBC, white blood cell; CRP, C-reactive protein; CRRT, continuous renal replacement therapy; MBP, mean blood pressure; HR, heart rate; CVP, central venous pressure; GRWR, graft-recipient-weight ratio. * The odds ratio of lymphocyte count-to-CRP ratio (<12.7) was a value obtained from another univariate and multivariate logistic regression model without the lymphocyte count-to-CRP ratio (continuous).

## Data Availability

The data presented in this study are available on reasonable request.
